# Paper 2: a systematic review of narrative therapy treatment outcomes for eating disorders—bridging the divide between practice-based evidence and evidence-based practice

**DOI:** 10.1186/s40337-022-00636-4

**Published:** 2022-09-12

**Authors:** Janet Conti, Lauren Heywood, Phillipa Hay, Rebecca Makaju Shrestha, Tania Perich

**Affiliations:** 1 School of Psychology, Western Sydney University, Sydney, Australia; 2 Translational Health Research Institute, Western Sydney University, Sydney, Australia; 3 School of Medicine, Western Sydney University, Sydney, Australia

**Keywords:** Eating disorders, Narrative therapy, Treatment outcomes, Systematic review

## Abstract

**Background:**

Narrative therapy has been proposed to have practice-based evidence however little is known about its research evidence-base in the treatment of eating disorders. The aim of this study was to conduct a systematic review of the outcome literature of narrative therapy for eating disorders.

**Method:**

Treatment outcome data were extracted from 33 eligible included studies following systematic search of five data bases. The study is reported according to Preferred Reporting items for Systematic Reviews and Meta-Analyses guidelines.

**Results:**

Of the identified 33 studies, 3 reported positive outcomes using psychometric instruments, albeit some were outdated. Otherwise, reported outcomes were based on therapy transcript material and therapist reports. The most commonly reported treatment outcome was in relation to shifts in identity narratives and improved personal agency with a trend towards under-reporting shifts in ED symptoms. Some improvements were reported in interpersonal and occupational engagement, reduced ED symptoms, and improved quality of life, however, there was an absence of standardized measures to support these reports.

**Conclusions:**

This systematic review found limited support for narrative therapy in the treatment of eating disorders through practice-based evidence in clinician reports and transcripts of therapy sessions. Less is known about systematic treatment outcomes of narrative therapy. There is a need to fill this gap to understand the effectiveness of narrative therapy in the treatment of EDs through systematic (1) Deliveries of this intervention; and (2) Reporting of outcomes. In doing so, the research arm of narrative therapy evidence base will become more comprehensively known.

**Supplementary Information:**

The online version contains supplementary material available at 10.1186/s40337-022-00636-4.

## Plain English summary

Narrative therapy has been proposed as a promising intervention for the treatment of eating disorders. However, the treatment outcomes of narrative therapy for eating disorders are under-researched. This systematic review of the literature has demonstrated limited support for narrative therapy through practice-based evidence in clinician reports and transcripts of therapy sessions. These reports demonstrated how narrative therapy was associated with identity shifts, some symptom reduction, reduced hospitalisations, improved agency over the problem and improvements in quality of life. There is a need for future research to systematically report treatment outcomes. This will fill a gap in research evidence-base for narrative therapy in the treatment of eating disorders.

**Supplementary Information:**

The online version contains supplementary material available at 10.1186/s40337-022-00636-4.

## Background

Eating disorders (EDs) such as anorexia nervosa (AN), bulimia nervosa (BN), binge eating disorder (BED) and other specified feeding and eating disorders [1] are prevalent in the community and have implications for physical, psychological and social wellbeing. Around 8.4% of women and 2.2% of men are diagnosed in their lifetimes [2] and, due to the nature of these conditions, EDs may be difficult to treat and often involve complex, ongoing care and multiple forms of treatment in both inpatient and outpatient settings [3].

Despite the prevalence and potential to run a chronic course that is associated with adverse impacts on quality of life [4], the effectiveness of the current evidence-based ED treatments is incomplete. Cochrane and other systematic reviews have shown that family therapies for AN, including Family-Based Therapy [5] and psychosocial treatments for BN, including cognitive behavioural therapy (CBT) and interpersonal psychotherapy [6] to be most efficacious treatments for these eating disorders, respectively. For example, systematic reviews have found that CBT for BN had moderate to large treatment outcome effects that were maintained over time [7] and benefits have been reported for adult AN [8]. There is less data to support the long term benefits of psychological therapies in the treatment of BED, however moderate support has been found for CBT and guided self-help [9]. Futhermore, a range of rates of relapse in EDs have been reported, as wide as from 9 to 52% [10], and definitions of relapse and remission rates may vary greatly within the literature.

There has also been increased research into the perspectives of those with a lived experience that goes some way in understanding how and why first line ED treatment do not work for all [11–13]. This has led many to suggest that there may be some sub-types of EDs, ‘severe and enduring’ in nature, that may require more specialised and targeted treatment over a longer period of time [14]. Others have suggested that treatments need to expand beyond a primary focus on eating behaviour change, for example to the rebuilding of identity outside the ED identity [12]. Furthermore, expanding the range of ED treatments that may be tailored to the experiencing person may go some way in preventing EDs running a chronic course and reduce the current rates of treatment attrition [12, 15, 16].

For those with severe and enduring EDs, there have been three randomised controlled trials to assess the effectiveness of treatment interventions [3]. Authors noted that although inpatient programs were found to be potentially effective in the shorter term, no evidence was found for longer term treatment gains for this group [3]. Types of treatments and settings may not necessarily be more effective than others, with insufficient evidence being found on one review for the superiority of either inpatient or outpatient settings [17].

Qualitative research of women who have recovered from eating disorders have noted that women experienced a fragmented sense of self when recovering, including rebuilding a more durable sense of identity with reclaiming relationships and self-acceptance also featuring as important [18]. Narrative therapy is a form of therapy developed by Michael White and David Epston [19–21] that provides an alternative therapeutic intervention that positions the person as the expert of their life and the problem (including an ED) as external to them. Focusing on identity and its performance, narrative therapy is a process-orientated therapy that focuses on externalizing and unpacking the meaning of problem stories to find and reconstruct hidden identity narratives that have been obscured by the dominant problem narrative [19]. This form of therapy is based on the philosophies of post-structuralism and social constructivism [21] and therefore understands that the language used in therapy matters in how identity narratives are constructed. In narrative therapy, therapists prioritise the language used by the person, including the metaphors to depicted their lived ED experience [20, 22], and proposes that language shapes identity narratives whose meaning are performed in a person’s life [19, 23].

In sum, narrative therapy may hold specific benefits in that it may address elements less considered in other therapies, such as the narrative metaphor to understand identity negotiation and its performance, which may fit the needs and preferences for the treatment of EDs as noted by those with a lived experience [12]. Narrative therapists have proposed that narrative therapy has “practice-based evidence” [24] for its effectiveness, however, to our knowledge there is no systematic review evaluating the research evidence for its efficacy in the treatment of EDs.

### The current study

The aim of this study was to conduct a systematic review of the literature to assess the reported therapeutic outcomes of narrative therapy in the treatment of ED. This review aims to determine the efficacy of narrative therapy interventions in the treatment of ED and to assess symptom measures and other reported treatment outcomes. A further aim of this review is to describe the range of treatment outcome variables, which have used in the systematically reviewed studies.

## Methods

### Design

This systematic review was carried out as per the guidelines set by the Preferred Reporting Items for Systematic Reviews and Meta-Analyses (PRISMA) [25].

The protocol is registered with PROSPERO and is available online: https://www.crd.york.ac.uk/prospero/display_record.php?ID=CRD42020175507.

### Identification and selection of studies

The databases searched included PsychINFO, MEDLINE, EMBASE, SocIndex and ProQuest Dissertations and Theses (grey literature) between 1979 and 4th July 2021. Key words used were (anorexi* OR anore*) OR (bulimi* or bulim*) OR (eat* or eating) OR (binge eat*) AND (intervention* OR treatment* OR therapy OR counsel*) AND (narrative).

The inclusion criteria were papers that met the following criteria: (a) published in English, (b) focused on the content of narrative therapy interventions (including specific details of said content); (c) included a sample of individuals in treatment for any ED, with the exception of books that describe narrative therapy interventions and case studies that use illustrative examples. Articles were excluded if they were (a) review papers, (b) not published in English, (c) if full text was unavailable, or (d) did not describe therapy outcomes. Therapy outcomes included scales measuring symptom severity and those described in case studies.

### Study selection

One reviewer (LH) ran the identified search terms across all electronic databases, including grey literature. Another reviewer (JC) identified relevant articles from their personal library of narrative therapy resources. All texts were then combined and duplicates removed. The title and abstract of each paper were individually evaluated by two reviewers (LH and JC) for their adherence to inclusion criteria and any discrepancies were resolved by a third reviewer (PH). The full text of publications were obtained if they met criteria and any unavailable full texts were excluded. The first reviewer (LH) assessed eligibility of full-text references for inclusion, with assistance from the second reviewer (JC) regarding any uncertainties.

Articles included were assessed at (i) Title screening, (ii) Abstract screening and (iii) Full text screening. Title, and abstract screening was independently undertaken by two reviewers (LH and JC). A third reviewer (PH) resolved any discrepancies. Full text screening was undertaken initially independently by two reviewers (LH and JC) and any discrepancies were resolved between them through discussion.

Articles that were selected for data extraction post full text screening included (i) An eating disorder (ii) Narrative therapy as a treatment of an eating disorder (iii) Full text available in English. Articles were excluded during full text screening included those that (i) Severely lacked any qualitative case study or quantitative data and; (ii) Theoretical papers. Data extraction was completed by LH.

### Quality assessment

All included publications were assessed independently by two reviewers (LH and JC) as outlined in paper 1 [26] using independent quality appraisal assessment tools adapted from the Downs & Black Checklist [27] and the Joanna Briggs Institute’s Checklist for Text and Opinion [28].

## Results

**Fig. 1:**
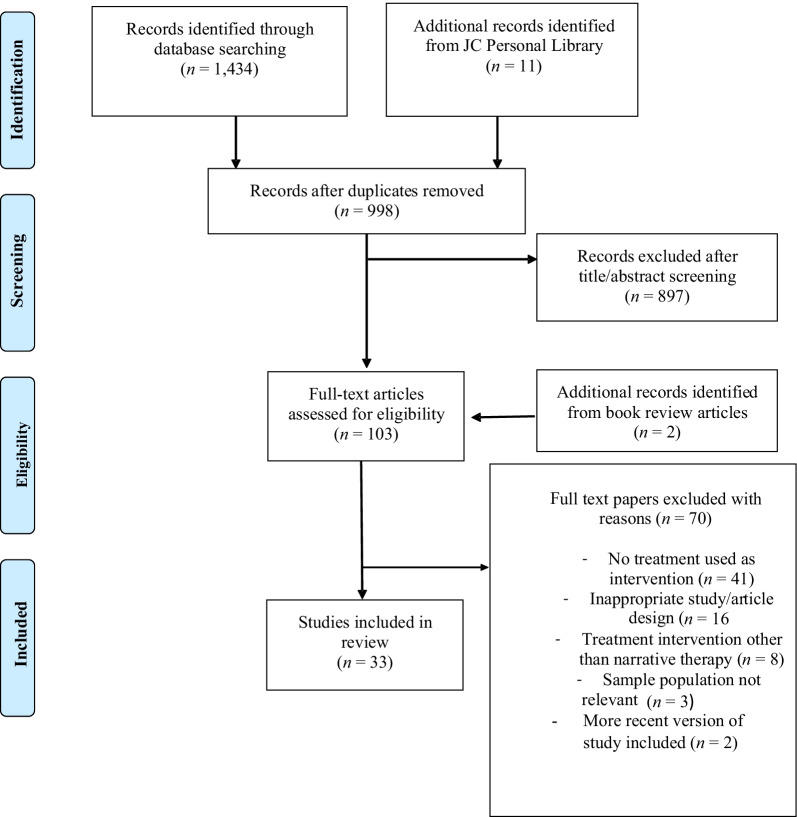
Flow chart of search strategy

The preliminary search from the combined databases yielded the results outlined in paper 1 [26] with 1434 results and an additional 11 articles from JC’s library. The same process was followed as outlined in flowchart for the search results in paper 1 [26] resulting in the same 33 texts included for this systematic review (Fig. 1). Fourteen of the 33 (42%) papers consisted of case study designs (*n* = 1), 10 (30%) case studies of between two to four clients, and six (18%) of the papers reported on five or more client cases. Three studies did not report the number of clients from which their data was obtained. Additional study characteristics are reported in paper 1 [26].

### Quality appraisal findings–outcome quality ratings

Table S1 and Table S2 (see Additional file 1) display the quality appraisal ratings for each of the included references. In relation to outcome data, seven (21%) articles clearly described the main outcomes of the intervention. Treatment outcomes were not reported in 8 (24%) papers and were unclear in 17 (52%) papers. The main findings were clearly reported in 14 (42%) papers and were unclear or not reported in the remaining papers. Overall, the quality ratings for the reporting of treatment outcomes indicated that these were insufficiently reported in the papers.

### Synthesis of narrative therapy outcomes

Three papers included quantitative outcome measures and two studies reporting ethics approval. All but one of the 32 papers reported qualitative outcomes, including participant experiences, therapy transcripts and therapist reflections (see Table S3 for data extraction summary, Additional file 1).

### Quantitative outcomes

Of the three studies reporting quantitative outcomes, one was a group narrative therapy intervention for seven participants with the dual presentations of eating concerns and depression [29]. The authors outlined that after a 10 week intervention, participants reported a reduction in ED symptoms as measured by the Eating Disorder Inventory (EDI) [30] and depressive symptoms as measured by the Depression, Anxiety and Stress Scale (DASS) [31]. They also reported results of a post-treatment survey that through externalisation and disengagement from the ED, participants reported a change in everyday living practices and less self-criticism.

A larger study with 645 participants in Israel reported that an intervention that integrated narrative therapy with motivational interviewing found that the dropout rate was < 10% during the first two months of treatment [32]. They also reported remission rates using the ED Global Clinical Score [33] of an average outcome score based on (i) Weight maintenance at least 15% ideal body weight; (ii) Menstruation in women for at least 12 months; (iii) Absence of purging behaviours; (iv) Normalization of eating habits; and (v) Social adjustment based on resumption of school or work. Reported remission rates at the end of treatment [15 months to 4 years]/4 year follow up were 69%/68% (AN) and 81%/83% (BN) respectively. Remission was defined as fully recovered or much improved, where much improved was defined as partial remission with infrequent occurrence of symptoms and return to social and occupational functioning.

A final study reported outcomes for a case study where a 28 year old woman had 10 sessions of narrative therapy over 12 weeks and reported a significant decrease on one of the scales of the EDI-3 (ascetism) [34] in addition to other qualitative therapist reported improvements.

### Qualitative outcomes

There were a range of qualitative outcomes that were cited by the papers as being reported by individuals who experienced narrative therapy for eating problems and some therapist reflections on what they learnt from those with a lived experience. This highlighted to two-way nature of the therapeutic relationship as highlighted by Michael White [35].

#### Client outcomes

A range of client outcomes were reported by authors of the included papers. Outcomes most frequently cited related to identity shifts and turning points over the course of therapy. Seventeen of the 33 papers outlined a range of ways that persons with a lived ED experience reclaimed and strengthened a sense of identity outside of the dominant ED identity [29, 36–38], including remembering who I am [39]; visualization of future without ED [40–47], improved confidence and self-esteem [48], self-expression [49], alongside reclaiming identity from abuse narratives [50, 51], and thickening preferred identity through values [52].

A number of narrative therapy practices were identified as key in the reclaiming of identities hidden by the problem saturated ED identity. A frequently cited practice was externalisation of the problem [29, 45, 47, 53–56] with the use of the person’s language forms or experience-near naming [45], including metaphor and personification of the problem [53, 54]. Other narrative practices that were associated with revealing identities concealed by the ED identity included: the tree of life metaphor [46] and the deconstruction of dominant discourses (or dismantling of taken-for-granted assumptions) that supported problematic identities [42, 47, 49, 55, 57].

Other outcomes reported included: reconnecting individuals with a sense of hope [36, 42, 46, 58, 59] and improvements in self-care and self-compassion [36, 39, 40, 44, 50, 51]. Furthermore, narrative therapy was also noted as facilitating individuals in the claiming of their voice [40, 58], including through their own speaking positions [60], and increased assertiveness [59, 61].

An increased sense of personal agency, including over the ED, was also noted as an outcome of the narrative therapy therapeutic process [29, 36, 38, 41, 43, 58, 61, 62] with two papers talking about how narrative therapy outcomes included increased readiness to change [58, 63]. Nine papers outlined a range of ways that individuals' eating practices were reported to have changed including reduced ED symptoms [29, 36, 40, 61], reduced hospital admissions [51, 59]; reduced fear about gaining weight [62], and discernment of body sensations related to eating [36] Three articles also reported improvements in associated mood and anxiety symptoms were evident over the course of the narrative therapy intervention [48, 51, 56].

Other treatment outcomes that were reported as having positive impacts on individuals’ relationships included emotional closeness [64], negotiating clearer boundaries in relationships [53, 65], connection with family and friends [39, 43, 51, 56, 59, 61] and others in the treatment [46]. Four papers also reported that the individuals resumed work and/or study after the narrative therapy treatment intervention [51, 56, 61, 65].

#### Therapist reflections–two way impacts of therapeutic intervention

Authors also outlined a range of ways that they were changed through their work as narrative therapists with those with a lived ED experience. This included a connection with their own creativity [39] and a recognition of the power imbued in professional contexts and the impacts of these on clients [60] and how therapy can also take the stance as political action to address injustices in society. Therapeutic letter writing was also seen as an opportunity for further reflection by both clients and therapists and also an opportunity for therapists to be open to being corrected by the client [64].

## Discussion

Narrative therapy outcomes to date are predominantly reported in the form of case studies with transcripts from therapy sessions and therapist reflections to exemplify some of the reported shifts in the context of therapy sessions. Three studies reported outcomes using standardized measures, including the EDI and DASS, with the largest study that included integrated narrative therapy with MI [32] measuring clinical outcome using the ED Global Clinical Score [33] that is currently outdated and infrequently used in current ED literature. These studies reported significant improvement in ED symptoms as measured by these instruments; however two of the studies had sample sizes of seven [29] and one respectively [34].

Given that identity shifts in the recovering of lost identities are a focus of narrative therapy, the most frequently cited treatment outcomes were stated on these terms. Examples of identity shifts were most frequently linked to the practice of externalisation of the problem with the use of the person’s own experience-near terms. Deconstruction or the unpacking of taken for granted assumptions supporting the ED identity was also cited as a narrative practice that facilitated the finding lost identities. Identity shifts, and addressing these in treatment, has been found to be an important component of ED treatment experiences and/or inadequately addressed in ED treatment interventions from the perspectives of those with a lived ED experience [12]. Narrative therapy has scope to comprehensively engage individuals (and their families) in finding hidden identity narratives that have been obscured by problem-saturated narratives or the ED identity. The papers included in this systematic review provided a range of exemplars of how narrative therapy practices engaged individuals in finding and strengthening hidden identity narratives.

Identity shifts in narrative therapy are understood as significant because they are not merely descriptive but also performative. For example, in the words of Jerome Bruner [23] “In the end, we *become* the autobiographical narratives by which we “tell about” our lives” (p. 694). Michael White & David Epston [21] have termed this enactment of identity narratives as performance of the meaning. Performing new meanings was noted in the therapy transcripts and therapist reports and included the areas of improved social and occupational functioning, some improvements in ED symptoms, and a reduction in need for hospital admissions. There was significant variability across the studies in the meaning performances that were reported. This may reflect the spirit of narrative therapy that is, the person is the expert of their life and the expert of what outcomes are significant to them. Nevertheless, the absence of consistency in the reporting of outcomes, including ED symptoms, means that the effectiveness of narrative therapy based on the research evidence is largely inconclusive.

The paucity of high quality quantitative research into narrative therapy is not unique to the treatment of EDs. Some of the broader challenges in researching the effectiveness of narrative therapy arise in the context of divergent philosophical paradigms. Psychotherapy research has traditionally assumed positivist epistemologies that require manualisation and replication of therapies [66]. These traditional research paradigms may be perceived to be at variance with the focus of narrative therapy on personal narratives, social and relational processes with a prioritization of the language and personal agency of the person with a lived experience [66, 67]. Nevertheless, narrative therapy research is gradually bridging the divide between positivist and social/relational approaches for example, in outcome research in depression [68] and post-traumatic stress disorder [69]. However, studies that focus on both outcomes and therapeutic process have continued to be predominantly exploratory [67].

Evidence-based practice has three arms: the research evidence and evidence from the clinician and the client’s experiences [70]. This review has found that there is limited support for narrative therapy for EDs from the practice-based evidence of clinician reports and therapy transcripts. However, in the absence of research that systematically analyses treatment outcomes, including in relation to ED symptoms, it is difficult if not impossible to know whether the narrative therapy intervention widely reported in a case study form will be translate to a broader group of individuals who experience EDs.

### Implications

This systematic review highlights how the outcomes of narrative therapy for EDs are currently under-reported and incomplete. However, this does not mean that narrative therapy interventions are ineffective in the treatment of EDs. Rather, it means that [1] there is an absence of systematic collection and analysis of treatment outcomes in terms of symptom improvement and quality of life, including social, relational and occupational engagement; and [2] further research is needed to document these and other outcomes that narrative therapists are witnesses to in their therapeutic practices.

In the absence of systematic reporting of outcomes, including relation to ED symptom reduction and improved quality of life, it is not possible at this point to conclude that narrative therapy is effective in the treatment of EDs. Narrative therapy continues to be positioned as having a “fringe role” [71] (p.77), including in the treatment of EDs. Being unrecognised as an “evidence-based practice” for eating disorders continues to limit narrative therapy practice in the treatment of EDs. This has implications for the breadth of treatment choices available to those with a lived ED experience.

There is a need for narrative therapists and researchers to engage in more systematic outcome research to substantiate what clinicians witness in the therapy room. Greater engagement is needed with the available tools being used to measure the outcome and effectiveness of treatments for EDs. If the existing tools are insufficient to measure the identity shifts and meaning performances that are central to narrative therapy, then new and more comprehensive tools need to be developed the prioritise the voice and personal agency for the experiencing person. Further research is also needed into ways that identity shifts mediate other changes, such as ED symptom reduction. Greater clarity is also needed as to the essential versus desirable components of narrative therapy and how outcome variables might be aligned and mapped onto these therapeutic components. Exploring outcomes through mixed methods design would provide scope to research narrative therapy outcomes in terms of both ED symptom reduction whilst also privileging the voice of the experiencing person in defining what recovery means to them. Given the understanding in narrative therapy that identity is constituted in socio-cultural discourses, identity shifts pre-, post, and within- therapy sessions would be well suited to analysis through critical discourse [72] and discursive [73] qualitative methodologies.

### Study strengths and limitations

In addition to the study’s strengths and limitations outlined in paper 1 [26], few of the papers in this review systematically reported on treatment outcome data, including ED symptoms. The majority of articles consisted of a clinician’s overall description of a treatment process with one to five clients. These papers included exemplar transcripts; however, this data was not analysed with qualitative methods. Likewise, very few of the papers used standardized medical or psychological measurements and when these were included, they tended to be outdated.

These omissions from papers on narrative therapy for EDs may be because narrative therapists position the experiencing person, rather than the therapist or researcher, as the expert on their life. This includes what therapeutic shifts are meaningful to the person and what constitutes recovery. Therefore the papers included these types of descriptions of outcomes of narrative therapy. This can be seen as both a strength of the papers and a limitation. A strength in that the papers gave voice to the person with a lived experience to determine what was significant for them in terms of treatment and shifts through therapy. A weakness in that there was an absence of consistency in the reporting of outcomes and an inability to draw conclusions about the effectiveness of narrative therapy in symptom reduction and improvements in quality of life.

Furthermore, there were no papers that reported a control group to compare outcomes between narrative therapy and another therapy or a control group. Because of the characteristics of the papers that met the study selection criteria, the quality assessment was based on text and opinion, and therefore descriptive. There was insufficient data in the papers to do a more comprehensive systematic review of treatment outcomes. Given the few papers that met selection criteria, we included all papers. This led to a bias towards greater inclusion of lower quality case studies by therapists that included their impressions of treatment outcome and selected exemplar transcripts to illustrate these.

### Concluding remarks

There are presently insufficient reports in the current literature to be able to make any conclusions or recommendations about the effectiveness of narrative therapy in the treatment of EDs. There is a need for researchers and practitioners to creatively engage in bridging the epistemological gap between positivist psychotherapy research and the practice-based evidence of clinicians who engage clients with narrative therapy. Consideration needs to be given to ways that narrative therapy interventions for EDs may be delivered, and their outcomes systematically measured, with a focus on social and emotional processes and without losing the spirit of narrative therapy where the person is positioned as not the problem and the expert of their life. This research has scope to be influential, not only in systematically researching outcomes for narrative therapy, but more broadly in the field of EDs where the experiencing person is at the centre of discerning what outcomes are significant for them and why, rather than this being decided primarily by researchers.

## Supplementary Information

**Additional file 1.** Narrative Therapy components and outcomes.

## Abbreviations

AN: Anorexia nervosa
BN: Bulimia nervosa
CBT: Cognitive behavioural therapy
EDI: Eating disorder inventory
DASS: Depression, anxiety and stress scale
